# Extractable Organofluorine Mass Balance Analysis of Aqueous Film-Forming Foam-Impacted Soils: Sample Pretreatment and a Combination of Target Analysis and Suspect Screening

**DOI:** 10.1021/acs.est.4c11909

**Published:** 2025-04-07

**Authors:** Qi Wang, Patrick van Hees, Patrik Karlsson, Enmiao Jiao, Marko Filipovic, Paul K. S. Lam, Leo W. Y. Yeung

**Affiliations:** † State Key Laboratory of Marine Pollution and Department of Chemistry, City University of Hong Kong, Tat Chee Avenue, Kowloon, Hong Kong 999077, China; ‡ Man-Technology-Environment (MTM) Research Centre, School of Science and Technology, Örebro University, Örebro 701 82, Sweden; § Eurofins Food & Feed Testing Sweden AB, Lidköping 531 40, Sweden; ∥ Key Laboratory of Yangtze River Water Environment, College of Environmental Science and Engineering, Tongji University, Shanghai 200092, China; ⊥ Niras Sweden AB, Hantverkargatan 11B, Stockholm 112 21, Sweden; # Department of Applied Science, School of Science and Technology, Hong Kong Metropolitan University, Hong Kong SAR 999077, China

**Keywords:** suspect screening, unknown organofluorine, source zones, zwitterionic and anionic PFAS, enhanced extraction, PFOS, PFHxS, FTAB

## Abstract

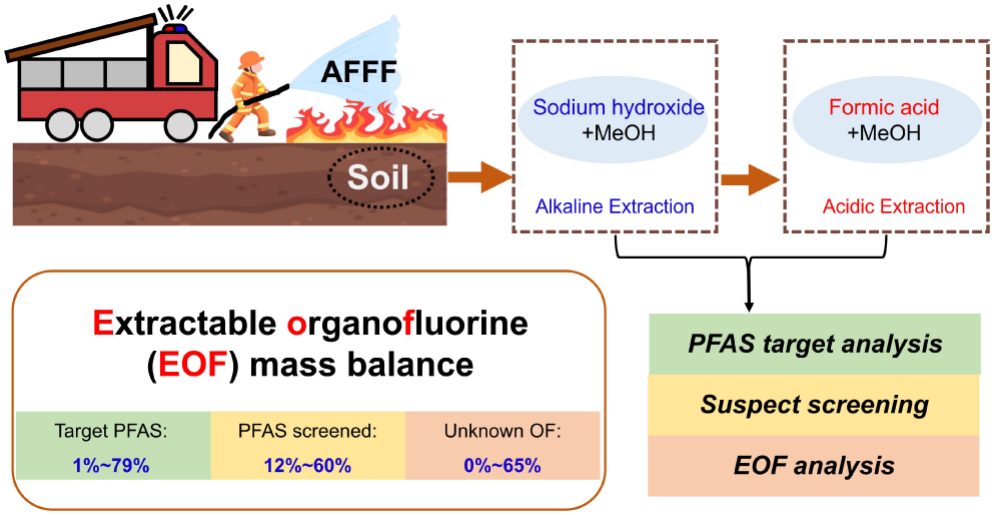

The application of aqueous film-forming foams (AFFFs) has caused considerable per- and polyfluoroalkyl substances (PFAS) pollution in the environment. Soil serves as a long-term source of PFAS for the adjacent groundwater and surface water, but the lack of extractable organofluorine (EOF) mass balance data in the AFFF-impacted soils may lead to an underestimation of PFAS contamination. This study analyzed ten surface soil samples from three AFFF-impacted sites in Sweden, using alkaline extraction followed by acidic extraction. The alkaline and acidic fractions were subjected to further cleanup and analyzed separately for target, suspect screening, and EOF analysis to evaluate the extraction efficiencies of different PFAS in the soil samples and reveal PFAS remaining unknown in the AFFF-impacted soils. Total target PFAS concentrations ranged from 33.0 to 2.40 × 10^4^ ng/g dry weight. Thirty-six PFAS were identified using suspect screening. Considerable amounts of zwitterionic and cationic PFAS (up to 58%) were identified in the acidic extraction fraction, while >95% of anionic PFAS were found in the alkaline extraction fraction. EOF mass balance analysis was conducted on AFFF-impacted soils for the first time. The high proportion of unexplained organofluorine (up to 65%) indicated the necessity for future investigation of the unknown PFAS in AFFF-impacted soils to comprehensively understand their fate and risk.

## Abstract 2

EOF mass balance was conducted for AFFF-impacted soils for the first time through target analysis, suspect screening, and EOF analysis. A significant proportion of UOF highlighted the necessity for further investigation into unknown PFAS in the AFFF-impacted soils.

## 1 Introduction

Per- and polyfluoroalkyl substances (PFAS) are synthetic fluorinated substances containing at least one fully fluorinated methyl or methylene carbon atom.^1^ PFAS comprise a vast array of compounds with distinct properties.^2^ Due to their excellent surface activity and exceptional biochemical stability, PFAS have been widely applied in numerous industrial and commercial applications for over 70 years.^3,4^ The strong C–F bonding strength (485 kJ/mol) of PFAS accounts for their incredible stability, resulting in their ubiquitous use worldwide.^4,5^ Among the vast number of PFAS, perfluoroalkyl acids (PFAAs), a class of anionic PFAS, have gained considerable attention in the last 20 years due to their highly persistent, bioaccumulative, and toxic properties, and their long-range transport potential.^4,6^ As a result, some PFAAs are (or proposed to be) listed under the Stockholm Convention as persistent organic pollutants for global elimination or restriction.^7^

Among the various sources of PFAS in the environment, the release of aqueous film-forming foam (AFFF) is particularly important.^8,9^ The complex composition and considerable usage of AFFF have resulted in high diversity and levels of PFAS contamination in AFFF-impacted sites (e.g., around military bases and airports).^8−12^ In addition to commonly studied anionic PFAS (e.g., PFAAs), substantial polyfluorinated substances, which are cationic and zwitterionic under environmental conditions, are also commonly identified in AFFF-impacted sites.^8,9,12^ For example, 6:2 fluorotelomer sulfonamidopropyl betaine (6:2 FTAB, also referred to as 6:2 FTSA-PrB) and related byproducts are frequently found.^11,12^ These cationic and zwitterionic PFAS were usually found to be at high concentrations in source zone surface soils under AFFF contamination, and the electrostatic interactions with negatively charged soil constituents are believed to be the main reason for the high resistance of zwitterionic and cationic PFAS in the soil.^13−18^ Such strong sorption makes commonly used soil extraction methods (i.e., alkaline extraction) insufficient for the extraction of zwitterionic and cationic PFAS in the soil, and an additional extraction step (e.g., acidic extraction) was needed.^9,19,20^ Moreover, unlike the strong environmental persistence of PFAAs, the nonfluorinated moiety attached to the perfluorinated chain implied that cationic and zwitterionic PFAS could transform to intermediate environmental degradation products and eventually to terminal PFAS (i.e., PFAAs) in the soil environment.^21,22^ The composition of PFAS in AFFF is complex, and their reference standards are usually not available, especially for the cationic and zwitterionic PFAS. This limited the comprehensive investigation of PFAS released from AFFF contaminations. High-resolution mass spectrometry (HRMS), such as quadrupole time-of-flight mass spectrometry (QToF-MS), is a useful technique for analyzing a broader suite of PFAS in the absence of reference standards.^23^ Through proving accurate masses, isotopic distributions, and MS/MS spectra, HRMS could elucidate PFAS composition and structural information in the AFFF and its impacted environmental samples.^24,25^ In addition, HRMS could semiquantify PFAS in the samples through their signal intensities (i.e., peak area) in the instrument without native standards. Currently, HRMS has been widely applied in investigating the composition and environmental fate of AFFF and its impacted environmental matrices (e.g., groundwater and soil).^9,24,26−28^ However, HRMS is unable to provide the total amount of PFAS, and other complementary analytical approaches must be used to assess how much PFAS remains in the samples.^29^

Considering the limited natural sources of organofluorines in the environment, most organofluorines present in the environment are likely to originate from anthropogenic sources.^30,31^ By conducting mass balance analysis of extractable organofluorine (EOF), the amount of unknown organofluorine (UOF) could be estimated, and the proportion of PFAS that remains unknown could be revealed. Previous EOF mass balance studies on AFFF indicated that the vast majority of PFAS in AFFF are known, but about half of OF remains unknown in the AFFF-impacted aquatic environment.^26,32,33^ The soil environment serves as a sink for PFAS in the AFFF source zone areas, but soil also acts as a long-term PFAS source to the adjacent aquatic environments (e.g., groundwater and surface water).^34−36^ In addition, the complex soil matrix and its active microbial activity provide a favorable environment for PFAS transformation, potentially leading to the formation of unidentified PFAS.^37^ Nevertheless, there are no reports regarding EOF mass balance in AFFF impacted soils to date, preventing us from understanding the amount of unknown PFAS and potentially leading to an underestimation of PFAS pollution in the AFFF-impact zones.

In light of this, a comprehensive extraction (i.e., basic and acidic extraction combined) and analysis were conducted on ten soil samples collected from three AFFF-impacted sites in Sweden. Different analytical approaches, including EOF analysis, target analysis, and HRMS screening, were applied to these soil samples to better understand the PFAS contamination status. The objectives of this study were to (1) investigate the EOF and PFAS pollution profiles in soil samples collected from AFFF-impacted regions in Sweden, (2) evaluate the extraction efficiencies of different PFAS in the soil samples using target and suspect screening approaches, and (3) reveal how much PFAS remains unknown in the studied AFFF-contaminated soil samples through the EOF mass balance.

## 2 Materials and Methods

### 2.1 Chemicals and Sampling

All analytical standards were purchased from Wellington Laboratories Inc. (Guelph, Canada). Detailed information on the studied PFAS is presented in Table S1. All standard solutions were prepared in HPLC-grade methanol. For EOF analysis, the PFOS standard used was from Sigma-Aldrich.

Ten soil samples were collected from three AFFF-impacted sites in Sweden. The sample identities were anonymized as sites A (*n* = 5), B (*n* = 4), and C (*n* = 1). Site A was an older AFFF training site that has not been used for more than 20 years. Sites B and C are still in use. No specific information is available about the formulations of the foams or any changes in their composition over time. The sampling test pits within the investigated sites included locations associated with the handling and storage of AFFF, areas subjected to firefighting training activities, and sites where fire truck cannon test shootings were conducted.

### 2.2 Sample Treatment

#### 2.2.1 Target Analysis

The sample treatment method for PFAS target analysis follows previous studies.^26,38,39^ Soil samples were freeze-dried and homogenized prior to analysis. Two nanograms of mixed mass-labeled surrogate standards were added to 0.1–1 g soil samples, followed by the addition of 7 mL of basic methanol (containing 0.2 M sodium hydroxide). The mixtures were vortexed for 30 s, sonicated for 30 min, and then separated by centrifugation at 5000 rpm (room temperature) for 5 min. Supernatants were transferred to a second 15 mL polypropylene tube. The extraction procedure was repeated once. After the second extraction round, two more rounds were performed in which the basic methanol was replaced with acidic methanol (containing 0.5 M formic acid). The basic and acidic extracts were concentrated to ∼0.5 mL under high-purity nitrogen.

The basic and acidic extracts were diluted with 10 mL of ultrapure water and adjusted to pH = 4 and 10 with formic acid and sodium hydroxide solution, respectively, for further SPE cleanup. For the basic extract, they were then loaded onto Oasis Weak Anion Exchange (WAX) cartridges, which were preconditioned by 4 mL of methanol with 0.1% NH_4_OH, 4 mL of methanol, and 4 mL of ultrapure water. When loading was finished, 4 mL of an ammonium acetate buffer solution (pH = 4) was used as the washing step, and the target analytes were eluted with 4 mL of 0.1% ammonia in methanol. The eluents were concentrated to 1 mL under high-purity nitrogen for PFAS target analysis. The acidic extracts were loaded onto the Oasis Weak Cation Exchange (WCX) cartridge, which was preconditioned with 4 mL of methanol with 0.1% formic acid, 4 mL of methanol, and 4 mL of Milli-Q water. 4 mL of 0.1% formic acid in methanol was used for elution, and the eluents were concentrated to 1 mL under high-purity nitrogen. Considering that the WAX cartridges may not fully capture zwitterionic and cationic PFAS in the water, the water that passed through the WAX cartridges during the cleanup of the basic extract was collected during the sample loading step and treated with the same procedure as the acidic extract. The concentrations of target PFAS were corrected by using the corresponding internal standards. A workflow for sample extraction and analysis in this process can be found in Figure S1.

#### 2.2.2 EOF Analysis and Suspect Screening

The extraction procedure for EOF analysis and suspect screening was similar to the PFAS target, but mass-labeled standards were not added to avoid the introduction of organofluorine. The eluents were evenly divided into two portions. One was directly used for EOF analysis, while the other was used for HRMS analysis after adding 1 ng of mixed mass-labeled internal standards and adjusting the volume to 0.5 mL.

#### 2.3 Instrument Analysis

All of the instrument analysis methods followed our previous studies. Details of the analysis methods can be found in the Supporting Information and elsewhere.^40−42^

##### 2.3.1 Target Analysis

The target compounds were analyzed using the Acquity UPLC system coupled with a Xevo TQ-S tandem mass spectrometer (Waters Corporation, Milford, USA) operated in negative electrospray ionization (ESI^–^) mode. Chromatographic separation was accomplished by using an Acquity BEH C18 column (2.1 mm × 100 mm, 1.7 μm) (Waters Corporation, Milford, USA). A gradient mobile phase of (A) 2 mM ammonium acetate in 30:70, methanol:Milli-Q (B) 2 mM ammonium acetate in methanol at a flow rate of 0.30 mL min^–1^ was used.

##### 2.3.2 Suspect Screening

Suspect screening was performed by using an Acquity UPLC system coupled with a quadrupole time-of-flight mass spectrometer (QToF) (G2-XS, Waters Corporation, Milford, USA) in the ESI^–^ and positive electrospray ionization (ESI^+^) modes, respectively. The LC conditions for suspect screening were the same as those used for target analysis, except that 0.1% formic acid in methanol was used as mobile phase B in the ESI^+^ mode. A data-independent acquisition (DIA) mode (i.e., MS^E^) was used to obtain the precursor and fragment ions. The confidence levels (CLs) were assigned following Charbonnet et al.^43^ The peak areas of the identified PFAS were normalized using internal standards with the closest retention time.

##### 2.3.3 EOF Analysis

The EOF was analyzed by combustion ion chromatography (CIC) (Metrohm, Switzerland). All fluorine was converted to hydrogen fluoride and absorbed into the water after combustion at 1000–1050 °C, and the dissolved fluoride was then analyzed by using the ion chromatograph. Quantification of the EOF was based on external calibration using PFOS as the standard that was combusted in the same way as the samples.

#### 2.4 Quality Assurance and Quality Control

Two procedural blanks, comprising empty vials, were analyzed alongside every batch of 10 samples. For target analysis, PFAS were quantified based on an internal calibration method using corresponding isotope-labeled internal standards. The method quantification limit (MQL) was determined as average concentrations in procedural blanks plus three times the standard deviation. The lowest point of the calibration curve that showed a signal-to-noise ratio greater than 10 was used as the MQL if the analyte was not found in the procedural blanks (Table S2). Recoveries of mass-labeled PFAS standards spiked in the soil sample before extraction (*n* = 3) ranged from 53 to 102% (Table S2).

For the suspect screening, procedural blanks were analyzed together with the samples. Only the peaks with intensity >10 times the intensity in the procedural blank were kept. The HRMS analysis results were compared to a suspect list that included AFFF-related PFAS reported in recent years (409 PFAS in negative mode and 344 PFAS in positive mode), and mass errors were set to 5 ppm.^24,41,44−46^

#### 2.5 Semiquantification and EOF Mass Balance

The semiquantification process followed the method described in a previous study.^47^ The anionic PFAS identified by suspect screening were semiquantified against structurally similar authentic standards based on the peak areas.^47^ The concentration of 6:2 FTAB in the analyzed soil samples agreed with what has been determined by an accredited commercial laboratory using the 6:2 FTAB authentic standard. The concentrations of other cationic PFAS were then calculated based on the ratios of their respective peak areas to the peak area of 6:2 FTAB and the known concentration of 6:2 FTAB.

To evaluate the amount of UOF, nonrecovery corrected PFAS concentrations of all target and suspect analytes (ng/mL PFAS) were converted to respective fluoride concentrations (ng/mL F). More details of the calculation are provided in the Supporting Information.

## 3 Results and Discussion

### 3.1 EOF and Target in the Alkaline and Acid Extraction Fractions

#### 3.1.1 Alkaline Extraction

In the basic extracts of the AFFF-contaminated soil samples from Sweden, EOF concentrations ranged from 73 (B2) to 2.40 × 10^4^ (A2) ng F/g dry weight (dw), which varied from the three sites in the order of (median values): site A (6.44 × 10^3^ ng F/g dw) > site B (557 ng F/g dw) > site C (245 ng F/g dw) (Table S3). Reports on EOF concentrations in the AFFF-impacted soil samples were scant. In the present study, EOF concentrations (median: 1350 ng F/g dw) are much higher than soil from Chinese fluorine industry parks (31.0–209 ng F/g dw), indicating high OF pollution in the investigated AFFF-impacted soil samples.^48^ It is important to note that research on EOF in the soil matrix remains limited, and there is currently no standardized method for tracing EOF in soils. Differences in sample treatment methods can have a significant effect on the results of EOF analysis in soils.

Target PFAS concentrations ranged from 33.0 (B2) to 2.40 × 10^4^ (A4) ng/g dw (Table S3). However, their median concentrations in the three sites were close, in the order of site A (367 ng/g dw) > site C (248 ng/g dw) > site B (247 ng/g dw). PFOS was the predominant PFAS, accounting for 14– 90% (median: 71%) of target PFAS concentrations in the sampling sites. An initial EOF mass balance was performed by integrating the results of EOF analysis and PFAS target analysis. The findings revealed that 1% (A2) to 80% (A4) of EOF in the soil samples could be explained by target PFAS, with a median value of 27%. This is the first report evaluating the EOF mass balance in the AFFF-impacted soil samples, and the relatively high UOF (median: 73%) proportion in the soil samples is consistent with the water samples from AFFF-impacted aquatic environment from Japan (UOF: 66%–91%), Sweden (UOF: 42–58%), and the U.S. (UOF: 54–82%).^26,32,33^ Nevertheless, target PFAS in the present work could not explain the majority of EOF, because most PFAS in the AFFF are zwitterionic and cationic compounds.^24,28,32^ These compounds were usually detected in ESI^+^ mode and are not included in our target analysis list. Thus, an evaluation of ″nonanionic PFAS” (i.e., PFAS screened using ESI^+^ mode) and acid extraction is needed, which will be discussed in the following sections.

#### 3.1.2 Acid Extraction

In the acidic extracts, EOF concentrations ranged from <10 to 326 ng F/g dw. Combined with the alkaline extraction, the total EOF concentrations reached 204 to 2.42 × 10^4^ ng F/g dw in the investigated soil samples. Negligible (<5% compared to the basic extracts) target PFAS were found in the acidic extracts, indicating alkaline extraction could extract the majority of target PFAS in the contaminated soil.

**Figure 1:**
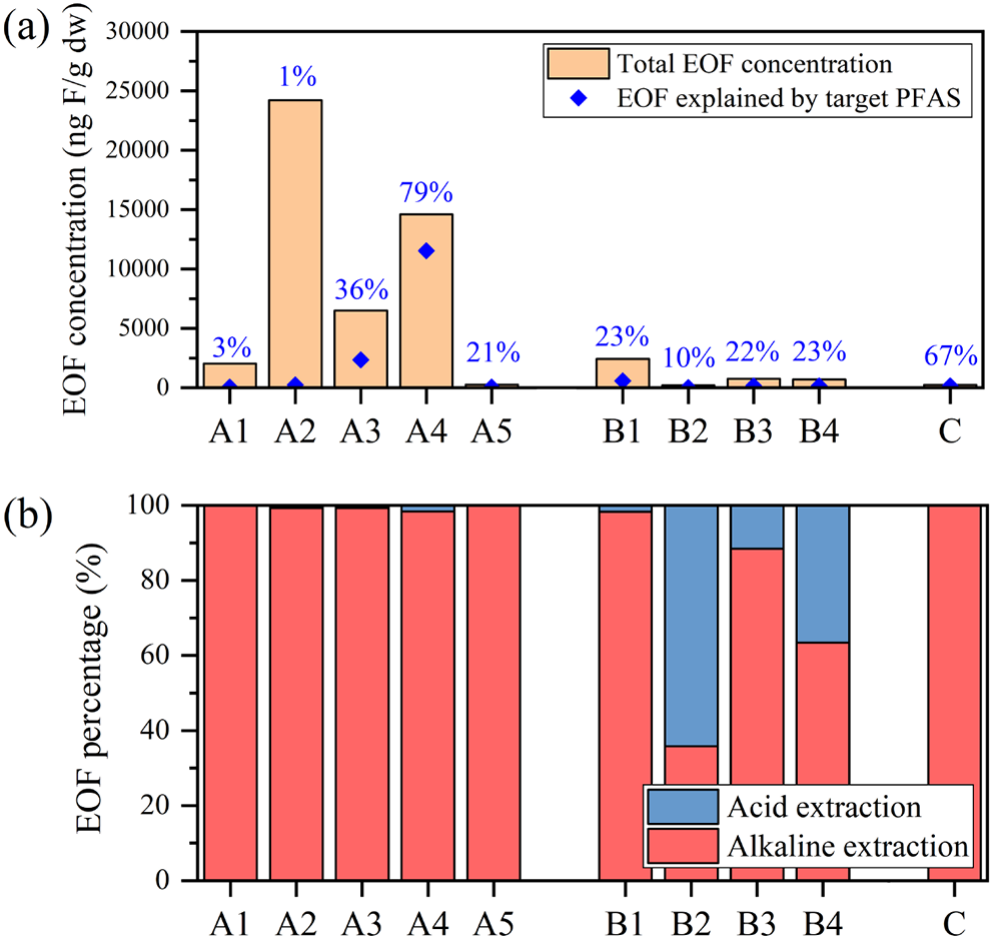
(a) EOF concentrations and EOF explained by target PFAS, with percentages on the bars indicating the contributions of target PFAS to EOF and (b) the composition of EOF in different extracts.

EOF in the acidic extracts accounted for less than 3% of the total EOF in the soil samples from sites A and C, indicating that most EOF was already extracted by alkaline extraction in these six soil samples (Figure 1). However, in the soil samples from site B, relatively high EOF concentration/composition was found in the acidic extracts (41–258 ng F/g dw, 2–64% of the total EOF were found in the acidic extract). This result indicates that if only alkaline extraction is used for tracing EOF in AFFF-impacted soil samples, the EOF levels in these samples may be underestimated. The variation in the EOF extraction efficiency in AFFF-impacted soil samples from different contaminated sites could be attributed to many factors, for example, PFAS pollution characteristics (composition and concentration) and the physical/chemical properties of the soil. This result implies that a more detailed analytical approach beyond target analysis (e.g., HRMS screening) is needed to investigate the UOF composition in the alkaline and acid extractions of the contaminated soils.

### 3.2 Suspect Screening of PFAS in the Alkaline Extractions

Suspect screening and semiquantification (SQ) analysis were conducted for both basic and acidic extracts through a suspect list used in previous studies to understand the composition and concentration of suspect PFAS candidates in both extracts.^24,44,45^Table 1 summarizes the PFAS identified via suspect screening at different CLs, and more details are listed in Tables S5 and S6. It should be noted that although some identified PFAS exhibited the same accurate mass, isotope distributions, and MS/MS fragmentation patterns as reported in the literature, their retention time patterns were not consistent with previous studies. This discrepancy could be due to the relatively complex structures of AFFF-related cationic PFAS, their potential transformations in soil, and coelution with co-extracted matrices during chromatographic separation, which might cause deviations in retention times. As a result, these PFAS were assigned a confidence level of 4 (CL4).^43^ The MS2 spectra for two most representative PFAS detected in this study—one from positive mode (6:2 FTAB) and one from negative mode (F5S-PFOS)—are shown in Figure S2. In total, 36 PFAS, 17 in the ESI^–^ mode and 19 in the ESI^+^ mode, from 17 classes were identified. An SQ was performed to evaluate the contribution of PFAS identified through suspect screening to the UOF that could not be fully explained by the target PFAS.

**Table 1:** PFAS Screened Using Suspect Screening

Name^a^	Formula	Observed *m*/*z*	Theoretical *m*/*z*	Mass error (ppm)	Retention time (min)^b^	CL^c^	Fraction	Sites
**ESI**^–^	[M–H]^−^							
H-PFOS	C_8_HF_16_O_3_S^–^	480.9394	480.9396	–0.51	7.78	2b	Alkaline	A, B, C
H-PFDS	C_10_HF_20_O_3_S^–^	580.9327	580.9332	–0.94	9.31	3b	Both	A, B, C
F5S-PFHxS	C_6_F_17_O_3_S_2_^–^	506.9019	506.9022	–0.68	8.77	2b	Both	A, C
F5S-PFHpS	C_7_F_19_O_3_S_2_^–^	556.8985	556.8991	–1.00	9.77	3b	Alkaline	A, B, C
F5S-PFOS	C_8_F_21_O_3_S_2_^–^	606.8950	606.8959	–1.42	10.40	2b	Both	A, B, C
F5S-PFNS	C_9_F_23_O_3_S_2_^–^	656.8912	656.8927	–2.24	10.82	3b	Alkaline	A, B, C
F5S-PFDS	C_10_F_25_O_3_S_2_^–^	706.8872	706.8895	–3.23	10.98	3b	Alkaline	B, C
E-PFNS	C_9_F_19_O_4_S^–^	564.9210	564.9219	–1.67	9.83	2b	Alkaline	A, C
K-PFOS	C_8_F_15_O_4_S^–^	476.9283	476.9283	–0.09	8.00	2b	Alkaline	A, C
K-PFUnDS	C_11_F_21_O_4_S^–^	626.9173	626.9188	–2.35	10.35	3b	Alkaline	B
K-PFDoDS	C_12_F_23_O_4_S^–^	726.9091	726.9124	–4.53	11.07	4	Alkaline	A, B, C
K-PFTrDS	C_14_F_27_O_4_S^–^	776.9064	776.9092	–3.61	11.40	3b	Alkaline	B
Cl-PFOS	C_8_F_16_O_3_ClS^–^	514.9018	514.9006	2.24	8.65	3b	Both	A, B, C
UPFOS	C_8_F_15_O_3_S^–^	460.9336	460.9334	0.34	7.79	3a	Alkaline	A, C
PFECHS^d^	C_8_F_15_O_3_S^–^	460.9338	460.9334	0.77	7.82	3a	Alkaline	A, C
PFHxSi	C_6_F_13_O_2_S^–^	382.9419	382.9416	0.67	7.28	2b	Alkaline	A, C
PFOSi	C_8_F_17_O_2_S^–^	482.9353	482.9353	0.07	7.97	2b	Alkaline	A, C
**ESI**^**+**^	[M + H]^+^							
4:2 FTAB	C_13_F_9_H_20_N_2_SO_4_^+^	471.1016	471.0994	4.67	8.08	2b	Alkaline	B
6:2 FTAB	C_15_F_13_H_20_N_2_SO_4_^+^	571.0933	571.0931	0.35	8.66	2a	Both	A, B, C
8:2 FTAB	C_17_F_17_H_20_N_2_SO_4_^+^	671.0859	671.0867	–1.19	10.20	2b	Both	A, B
10:2 FTAB	C_19_F_21_H_20_N_2_SO_4_^+^	771.0783	771.0803	–2.59	11.29	2b	Both	A, B
12:2 FTAB	C_21_F_25_H_20_N_2_SO_4_^+^	871.0749	871.0739	1.15	12.08	2b	Both	A, B
14:2 FTAB	C_23_F_29_H_20_N_2_SO_4_^+^	971.0696	971.0675	2.16	12.66	3b	Alkaline	A, B
N-HOEAmP-FHxSA	C_13_H_18_F_13_N_2_O_3_S^+^	529.0823	529.0825	–0.38	8.34	2b	Both	A, B
N-HOEAmP-FHxSE	C_15_H_22_F_13_N_2_O_4_S^+^	573.1077	573.1087	–1.74	8.40	4	Both	A, B
N-TAmP-N-FHxSA	C_13_H_18_F_13_N_2_O_2_S^+^	513.0888	513.0875	2.53	8.47	4	Both	A, B
N-TAmP-N-FOSA	C_15_H_18_F_17_N_2_O_2_S^+^	613.0819	613.0812	1.14	10.08	4	Alkaline	A, B
6:2 FTSHA-sulfoxide	C_14_F_13_H_19_NO_2_S^+^	512.0935	512.0923	2.34	8.10	2b	Both	A, B
N-TAmP-MeFHxSA	C_12_H_16_F_13_N_2_O_2_S^+^	499.0731	499.0719	2.40	8.47	2b	Both	A, B
N-TAmP-MeFOSA	C_14_H_16_F_17_N_2_O_2_S^+^	599.0665	599.0656	1.50	10.08	2b	Both	A, B, C
PFHxSaAm	C_11_F_13_H_14_N_2_O_2_S^+^	485.0572	485.0562	2.06	8.47	4	Both	A, B
PFOSaAm	C_13_F_17_H_14_N_2_O_2_S^+^	585.0504	585.0499	0.85	10.06	4	Alkaline	A
N-SPAmP-FHxSA	C_14_H_20_F_13_N_2_O_5_S_2_^+^	607.0589	607.0600	–1.81	8.51	4	Both	A, B
N-SPAmP-FOSA	C_16_H_20_F_17_N_2_O_5_S_2_^+^	707.0519	707.0537	–2.55	9.43	4	Alkaline	A, B
N-CMAmP-FHxSA	C_13_H_16_F_13_N_2_O_4_S^+^	543.0623	543.0618	0.92	9.84	4	Both	A, B
N-CMAmP-FOSA	C_15_H_16_F_17_N_2_O_4_S^+^	643.0558	643.0554	0.62	10.14	4	Alkaline	A, B

a The name of the PFAS in this work followed a previous study.^24^ The full name and structure were listed in Supporting Information.

b The reported retention time is the average retention time of the compound in detectable samples.

c CL = confidence level, which was assigned following Charbonnet et al.^43^

d The UPFOS and PFECHS have the same precursor *m*/*z* but are distinguished through the fragment relative abundance followed by a previous study.^24^

#### 3.2.1 ESI^–^ Mode

In ESI^–^ mode, 17 PFAS from 8 classes were identified (Table 1), including two hydro-substituted PFSA (H-PFSA), five pentafluoro sulfide PFSA (F5S-PFSA), ether perfluorononanesulfonic acid (E-PFNS), four ketone PFSA (K-PFSA), chlorinated PFOS (Cl-PFOS), unsaturated PFOS (UPFOS), perfluoroethylcyclohexane sulfonate (PFECHS), and two perfluoroalkanesulfinate (PFSAi). PFAS screened in the present work are all substituted PFAA derivatives, and they were reported to exist widely in the AFFFs.^24^ According to the SQ results, the total concentrations of PFAS screened in ESI^–^ (ΣSQ^–^) ranged from 2.7 (B2) to 144 (B1) ng/g dw, which were much lower compared with the total target PFAS concentrations (33.0 to 2.40 × 10^4^ ng/g dw). In sites A1–A5 and B1–B4, ΣSQ^–^ could explain 0–9% (median: 2%) of the UOF in the investigated soil samples, implying that SQ^–^ is not the main contributor to UOF in basic extracts in sites A and B. Nevertheless, in site C, about half (48%) of the UOF in basic extracts could be explained by ΣSQ^–^, which indicated different PFAS contamination profiles in different sites.

All PFAS, except F5S-PFHxS (C6), PFHxSi (C6), and F5S-PFHpS (C7), are long-chain PFAS (C ≥ 8), which could be explained by the relatively high binding ability with organic matter in these long-chain PFAS. F5S-PFSA is the predominant class in all the soil samples, which accounted for >85% of ΣSQ^–^ in sites A and B and 62% in site C. The F5S-PFSAs were regarded as derivatives of PFSAs and PFCAs in AFFF, and the similar structure between F5S-PFSAs and PFSA indicated that they have a similar environmental fate.^24,26^ K-PFSA was the second most prevalent PFAS class, contributing 0–8% to the ΣSQ^–^. Some K-PFSA analogues, including K-PFDoDS (C12) and K-PFTrDS (C13), were not reported in the AFFF samples before but were also found in the investigated contaminated soil samples. This result is consistent with a previous study where K-PFSAs were widely detected in AFFF-contaminated soil samples.^9^ The reason for this could be that these long-chain K-PFSAs originate from the degradation of some PFAS in the AFFF, which occurred in the soil environment.^21,22^

#### 3.2.2 ESI^+^ Mode

In ESI^+^ mode, 19 PFAS from 7 classes were identified, and all of these PFAS are ECF-derived sulfonamide-based compounds. The total semiquantified concentrations of PFAS screened in ESI^+^ (ΣSQ^+^) ranged from 19.5 (C) to 1.60 × 10^4^ (A2) ng/g dw, which were comparable to the total target PFAS concentrations (33.0 to 2.40 × 10^4^ ng/g dw). The ΣSQ^+^ contributed 22–90% (median: 38%) to the UOF in sites A and B. By combining the results of ΣSQ^+^ and ΣSQ^–^, about half of the UOF could be revealed (33–93%, median: 47%) in the alkaline extraction. Nevertheless, ΣSQ^+^ could only explain 9% of the UOF in site C, which is consistent with its relatively high SQ^–^ (i.e., anionic) PFAS pollution profile (ΣSQ^–^: 48% to total UOF).

In most sites (except for B3 and B4), X:2 FTAB was the predominant PFAS class, accounting for about half (40–91%) of the SQ^+^. In sites B3 and B4, *N*-trimethylammoniopropyl-perfluoroalkanesulfonamide (N-TAmP-FASA) was the predominant PFAS class, accounting for 60 and 69% of the SQ^+^ while FTABs only contributed 39 and 9% of the SQ^+^ in these two sites, respectively. These two classes of PFAS were also usually reported to widely exist in the AFFF-contaminated soil sample, usually being the predominant zwitterionic and cationic PFAS.^9,17,27^ Regarding the perfluorocarbon chain length, six X:2 FTAB congeners were detected, which ranged from 4:2 to 14:2, while 6:2 and 8:2 FTAB dominated in the X:2 FTAB class (average proportion: 57 and 21%, respectively). Apart from X:2 FTAB, all the other PFAS screened using ESI^+^ have a perfluorocarbon chain length of six and eight (i.e., C6 and C8 ECF compounds), which have the potential to transform into PFHxS and PFOS and possibly bring ecological risks.

### 3.3 Suspect Screening of PFAS in the Acidic Extractions

Most (13 out of 17) of the PFAS screened in the ESI^–^ mode were exclusively detected in the basic extract and not in the acidic extract. However, the remaining four PFAS, in addition to being detected in the basic extract, were also occasionally found in the acidic extract, with small proportions (<8%, based on SQ results) present. This result indicated that basic extraction fully extracted the anionic PFAS screened in this work, which is in line with the results on the anionic PFAS investigated through target analysis in this work. It should be noted that no additional PFAS were found in the acidic extract compared to the basic extract, which indicated that alkaline extraction might underestimate the amount of PFAS but does not lose the number of PFAS suspects in this work.

**Figure 2:**
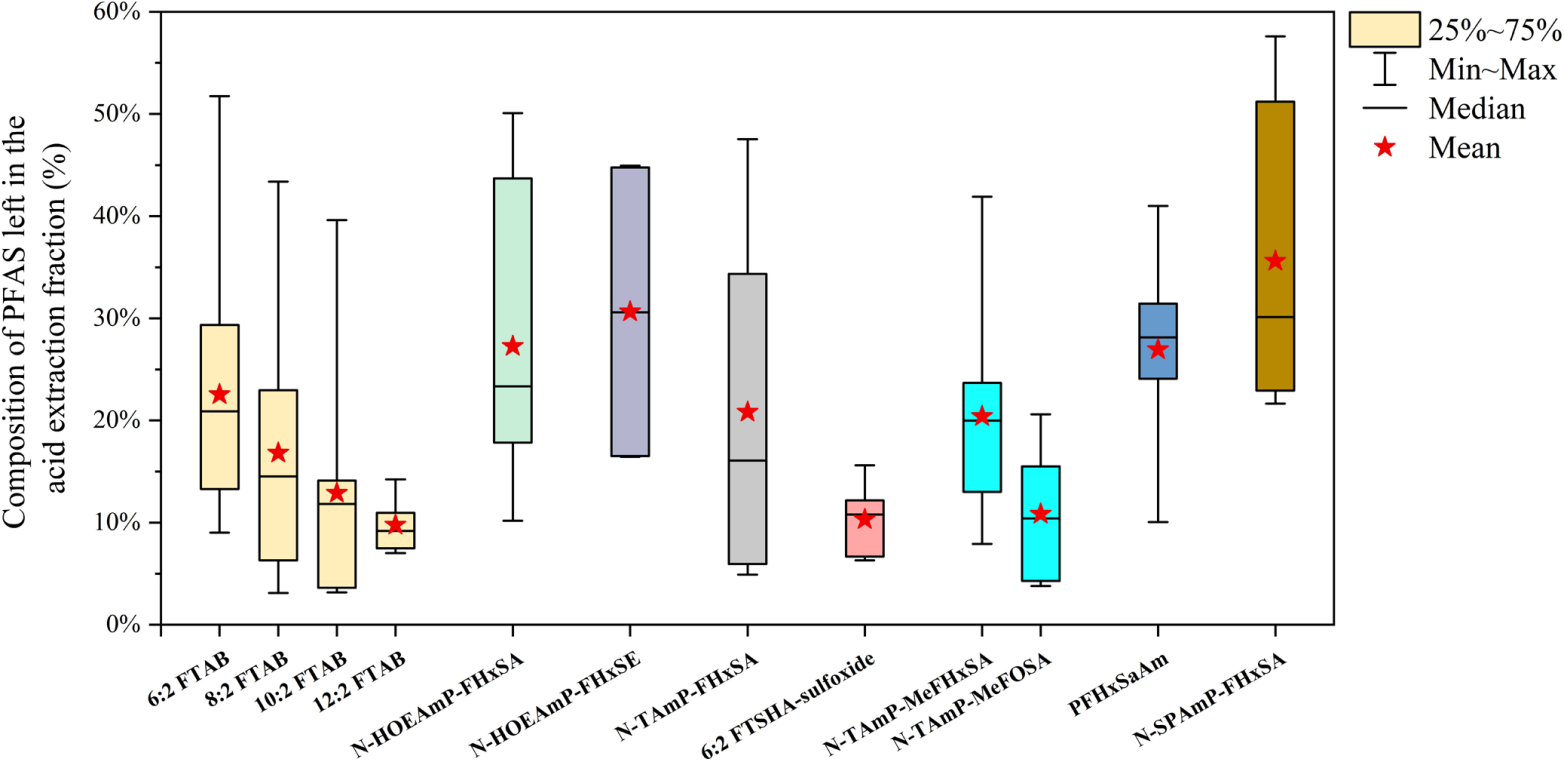
Proportion of zwitterionic and cationic PFAS remaining in the acidic extract.

In contrast to the alkaline extraction of anionic PFAS, 12 zwitterionic and cationic PFAS were present in the acidic extraction in at least four soil samples (Figure 2 and Table S7). The proportion of these PFAS left in the acidic extraction (*F*_acid_, %) was up to 58% (*N*-sulfopropyldimethylammonio propyl perfluorohexanesulfonamide in site B2) according to the SQ^+^ result. This result indicated that only using alkaline extraction could underestimate PFAS screened in the ESI^+^ in the AFFF-contaminated soils, which is consistent with previous studies showing that alkaline extraction is insufficient for the extraction of zwitterionic and cationic PFAS from soil samples.^9,20,24,49^ The inadequate extraction for zwitterionic and cationic PFAS using alkaline extraction could potentially be attributed to their enhanced sorption to soils, with the major sorption driving forces including hydrophobic effect, electrostatic interactions, and cation exchange.^16,24^ However, the sorption capacity of different PFAS to soil could be complex in the realistic environment, as the physical and chemical properties of soils (e.g., organic carbon content, anion-exchange capacity, and effective cation-exchange capacity) could highly influence the sorption capacity and affect the alkaline extraction efficiency (i.e., *F*_acid_ in this work) in the future.^9,16,24^

Four FTAB congeners, including 6:2, 8:2, 10:2, and 12:2 FTABs, were found in the acidic extraction, with median *F*_acid_ values of 21, 15, 12, and 9%, respectively, and a decreasing trend was found for the proportion of FTAB left after alkaline extraction. This result is contrary to a previous study where extraction efficiencies for shorter perfluorinated carbon chain zwitterionic and cationic PFAS in soil samples were reported to be better than their longer chain analogues.^24^ Studies have demonstrated that longer-chain PFAS analogues have a greater affinity for soil and sediment compared to shorter-chain analogues.^16,24,50^ However, the adsorption capacity of PFAS in the same soil is also affected by its concentration, and the distribution coefficient was found to decrease with an increase in the PFAS concentration.^16^ In the investigated soil samples, the median concentrations of 6:2, 8:2, 10:2, and 12:2 FTABs in basic extracts were 346, 107, 73.7, and 14.8 ng/g dw, respectively. The higher concentrations of the shorter chain FTABs could be an important reason for their higher residual in the soil after alkaline extraction compared to longer chain FTABs. Nevertheless, no significant positive correlation was found between the individual FTAB concentration and *F*_acid_ in different soil samples (*p* < 0.05). Moreover, electrostatic interactions with negatively charged soil constituents are another important factor in zwitterionic and cationic PFAS sorption on soil, which is more significant when the PFAS concentrations decrease.^16^ With the same charge but lower molecular weight, shorter chain zwitterionic and cationic PFAS could have a stronger electrostatic interaction capacity than the longer chain analogues, which could be another possible reason for the higher proportion of the shorter chain FTABs in the acidic extract.

A similar result was also observed for *N*-TAmP-FASA, where the *F*_acid_ of N-TAmP-FHxSA (perfluorocarbon chain length = 6, 20%) is higher than that of N-TAmP-FOSA (perfluorocarbon chain length = 8, 10%). In addition, for the other zwitterionic and cationic PFAS classes (except for 6:2 FTSHA-sulfoxide where no other analogues were detected), the 8:2 homologues could only be detected in the alkaline extract, while 6:2 homologues were found to widely exist in the acidic extract. This result indicated that alkaline extraction could miss more short-chain ECF zwitterionic and cationic PFAS than long-chain ones. Therefore, previous studies on zwitterionic and cationic PFAS in soil samples may overlook more 6:2 homologues compared to other homologues (e.g., 8:2 homologues), especially for the sites where 6:2 ECF zwitterionic and cationic PFAS are present at high levels in the soil samples.

### 3.4 EOF Mass Balance in the Contaminated Soil Samples Using the Comprehensive Extraction and Analysis Method

**Figure 3:**
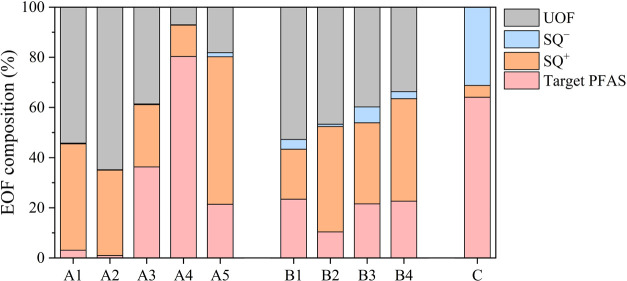
EOF mass balance through different analytical methods.

As mentioned above, a considerable amount of EOF was found to be left in the acidic extract, while SQ contributed a certain amount of EOF in the soil samples. Therefore, a combined EOF mass balance was used to consider basic and acidic extraction and target and subsuspect analysis in different soil samples (Figure 3). Detailed data can be found in Table S8.

In soil samples A1–A5, 35–93% of the EOF could be explained by target analysis and SQ approaches. Target PFAS, SQ^+^, and SQ^–^ accounted for 1–80%, 12–59%, and 0–2% of the EOF, respectively. In soil samples B1–B4, 47–66% of the EOF were revealed, while target PFAS, SQ^+^, and SQ^–^ accounted for 10–23%, 20–42%, and 1–% of the total known OF, respectively. A high amount (median: 34 and 37% for sites A and B, respectively) of EOF could be attributed to SQ^+^, indicating the significance of including these zwitterionic and cationic PFAS in the EOF mass balance in the AFFF-impacted soil samples. In these nine soil samples from sites A and B, about half (median: 40%) of the EOF still remained unknown, which could possibly be attributed to the potential (bio)transformation of PFAS precursors to the PFAS that were not included in the suspect list in this work. In order to characterize the UOF, a PFAS homologue-based nontarget analysis was conducted, following our previous work, to identify potential unknown PFAS homologues with a mass difference corresponding to −CF_2_– (49.99681 Da) or −CH_2_CF_2_– (64.01246 Da) units.^25^ Peaks were extracted and screened based on their CF_2_ mass defects, and only those with a mass defect >0.85 or <0.15 were retained. The peaks were also evaluated for an ascending trend of mass versus retention time, and only those meeting this criterion were listed as candidate homologues. However, no additional PFAS classes were observed to meet these criteria. Therefore, future nontarget screening in the AFFF-impacted soil samples is needed to comprehensively reveal the PFAS pollution profiles as these unknown PFAS may bring undiscovered risks.

In site C, virtually all EOF (104%) could be explained by target analysis and the SQ approach, and target PFAS, SQ^+^, and SQ^–^ accounting for 67, 5, and 32% of the EOF, respectively. This result indicated a different PFAS profile in site C compared with sites A and B. In sites A and B, about half (median value: 42%) of the known OF could be attributed to zwitterionic and cationic PFAS, while only 5% of the known OF in site C originated from the contribution of zwitterionic and cationic PFAS. Zwitterionic and cationic PFAS are less stable than most of the anionic PFAS investigated in this study (e.g., PFCAs, PFSAs, and F5S-PFSAs).^22^ Moreover, a significant positive correlation was found between the UOF proportion and the percentage of SQ^+^ contributed to the known OF (contribution of zwitterionic and cationic PFAS to total known OF) (Spearman’s rank correlation, *p* = 0.013, *r^2^* = 0.745, *n* = 10, Figure S3). This result indicated that an increase in the zwitterionic and cationic PFAS contribution could bring more UOF in the soil samples, and the relatively low zwitterionic and cationic PFAS (or high anionic PFAS) proportion in site C should be the main reason that most of the EOF could be explained by the target analysis and SQ approach in this site. Nevertheless, this result requires further verification as only ten AFFF-impacted soil samples were involved in this work, and the SQ approach may bring some uncertainty.

### 4 Environmental Implications

AFFF contamination has been a long-standing environmental problem. Among the AFFF-impacted environmental matrices, soil is crucial as it not only serves as a sink for PFAS in the source zone areas but also as a long-term PFAS source to groundwater and adjacent surface water. A comprehensive extraction (i.e., alkaline and acid extraction) method was used to investigate the occurrence of PFAS and EOF in the AFFF-impacted soil samples, and both quantification (LC-MS/MS-based target analysis) and SQ (HRMS-based suspect screening) approaches were applied. Considerable zwitterionic and cationic PFAS were found using SQ^+^, indicating that significant amounts of PFAS may be overlooked if ESI^+^ suspect screening is omitted from the analysis. In addition, the wide prevalence (13 substances) and relatively high proportion (up to 58%) of zwitterionic and cationic PFAS found in the acidic extract suggested the necessity of this additional extraction step (i.e., acidic extraction after alkaline extraction) to treat PFAS in the AFFF-impacted soils, which is consistent with previous studies.^9,19,20^ Nevertheless, no additional PFAS were found in the acidic extract compared to the basic extract in this work, implying that reliance on alkaline extraction alone may lose some amount of PFAS but not the number of “suspects”. The extraction efficiency of basic extraction on different PFAS is affected by many factors, for example, soil texture, PFAS concentration, and its own nature (e.g., cationic or anionic, perfluorocarbon chain length). For FTABs with a perfluorocarbon chain length from 6–12, a decreasing trend was found for the proportion of FTABs left after alkaline extraction. This may be attributed to the higher concentrations of the shorter chain FTABs compared to those of longer chain FTABs, implying the role of PFAS concentrations on extraction efficiency.

With the growing adoption of HRMS in recent years, suspect screening and SQ have been widely applied in studies of PFAS contamination. This study applied the EOF mass balance in AFFF-impacted soils for the first time, providing valuable insights to support the improvement of soil management and remediation strategies for PFAS-contaminated sites. Our results highlight the necessity of integrating EOF mass balance to ensure the comprehensive identification of PFAS, which is particularly critical in regions with high levels of PFAS pollution. Quantification and semiquantification approaches fail to explain substantial fractions of EOF (UOF proportion: up to 65% of EOF) in some AFFF-impacted soils, emphasizing the need for further research to better understand the fate and risks of unknown PFAS compounds in contaminated soils. This may be a result of the biotic/abiotic transformation of zwitterionic and cationic PFAS in the soil, and future PFAS nontarget screenings in the AFFF-impacted soil samples are warranted.

There are certain limitations in this study that warrant further investigation. To enhance detection sensitivity and achieve broader coverage of PFAS in AFFF-impacted soil samples with a complex matrix, a DIA mode was employed to obtain both precursor and fragment ions. However, this approach resulted in the MS2 spectra being partially influenced by other non-target ions during co-elution. The retention time patterns of certain cationic PFAS, assigned a confidence level of 4 in this study, were found to be inconsistent with those reported in the literature, despite exhibiting identical accurate mass, isotope distributions, and MS/MS fragmentation patterns. This discrepancy could potentially be attributed to transformations occurring in the soil, leading to structural modifications that differ from those described in prior studies.

In addition, the uncertainty in the results due to the use of semiquantification analysis cannot be ignored. The responses of different PFAS classes on HRMS can vary; however, the number of available authentic PFAS standards is limited compared to the diverse range of PFAS identified in AFFF and impacted environmental samples, especially for the zwitterionic and cationic PFAS detected in ESI^+^ mode. A more precise SQ method for PFAS determination should be developed when more authentic PFAS standards become available. Additionally, the integration of other analytical techniques (e.g., PFAS precursor oxidative conversion) is recommended to enhance the accuracy of SQ.^32,49^

## Supporting Information Available

*m*/*z* values used for suspect screening and their source (XLSX)
This section contains the following information: details of chemicals and instrumental analysis; matrix-spiked MQLs and recoveries; PFAS screened using suspect screening; diagnostic fragments and reference standards of PFAS screened using suspect screening; OF contribution from different proportion; a workflow for sample extraction and analysis; MS2 spectra for 6:2 FTAB in positive mode and F5S-PFOS in negative mode: and correlations between proportions of UOF and zwitterionic and cationic PFAS (PDF)
