# Supplementary material for: Extractable Organofluorine Mass Balance Analysis of Aqueous Film-Forming Foam-Impacted Soils: Sample Pretreatment and a Combination of Target Analysis and Suspect Screening
Source: Environ Sci Technol. 2025 Apr 7;59(15):7624–33. doi: 10.1021/acs.est.4c11909 (PMC12020414; doi:10.1021/acs.est.4c11909)
Supplement: Supplementary file 2 — es4c11909_si_002.pdf [file es4c11909_si_002.pdf]

Supporting Information (SI)

Extractable organofluorine mass balance analysis of aqueous film-forming foam-impacted soils: Sample pretreatment and a combination of target analysis and suspect screening

Qi Wang,[1,2] Patrick van Hees,[2,3] Patrik Karlsson,[3] Enmiao Jiao,[2,4] Marko Filipovic,[5,#] Paul K. S. Lam,[1,6] Leo W. Y. Yeung [2]*

[1]State Key Laboratory of Marine Pollution and Department of Chemistry, City University of Hong Kong, Tat Chee Avenue, Kowloon, Hong Kong 999077, China.
[2]Man-Technology-Environment (MTM) Research Centre, School of Science and Technology, Örebro University, 701 82 Örebro, Sweden.
[3]Eurofins Food & Feed Testing Sweden AB, 531 40 Lidköping, Sweden.
[4]Key Laboratory of Yangtze River Water Environment, College of Environmental Science and Engineering, Tongji University, Shanghai 200092, China.
[5]Niras Sweden AB, Hantverkargatan 11B, 112 21 Stockholm, Sweden.
[6] Department of Applied Science, School of Science and Technology, Hong Kong Metropolitan University, Hong Kong SAR 999077, China.

[#] Current address: Sellén & Filipovic AB, Odd Fellows gränd 1, 611 34 Nyköping, Sweden.

<u>**Corresponding author:**</u>
Leo.Yeung@oru.se.

Number of tables: 8
Number of figures: 3
Number of pages: 19

Table of Contents

	Page
Details on chemicals and instrumental analysis	S3
Details on conversion of PFAS concentration in F-equivalent concentration	S4
Tables	
Table S1. Mass spectrometric information for PFAS target analysis	S5
Table S2. Recoveries and MQL for PFAS target analysis	S6
Table S3. EOF concentrations in the alkaline and acid extractions	S7
Table S4. Target PFAS concentrations in the soil samples	S8
Table S5. Structure and the full name of PFAS screened using suspect screening	S9
Table S6 Diagnostic fragments and reference standards of PFAS screened using suspect screening	S12
Table S7. The proportion of PFAS left in the acid extraction	S14
Table S8. OF contribution from different proportion	S15
Figures	
Figure S1. A workflow for sample extraction and analysis	S16
Figure S2. MS2 spectra for 6:2 FTAB in positive mode and F5S-PFOS in negative mode	S17
Figure S3. Relationship between SQ^+ to total known EOF and UOF proportion in soil samples	S18
References	S19

Chemicals

All native standards, including perfluoroalkyl carboxylic acids (PFCAs; C4-C14, C16, and C18), perfluoroalkyl sulfonic acids (PFSAs; C4-C10 and C12), perfluoroalkyl sulfonamides (FASA; C6 and C8), and fluorotelomer sulfonic acids (FTSAs; 4:2, 6:2 and 8:2) were purchased from Wellington Laboratories (Guelph, ON, Canada).

Instrumental analysis

The instrumental analysis applied in this work follower our previous studies.[1-3]

PFAS target compounds were analyzed using the Acquity UPLC system coupled with the Xevo TQ-S tandem mass spectrometer (Waters Corporation, Milford, USA) operated in the electrospray negative ionization mode. The chromatographic separation was accomplished by using an Acquity BEH C18 column (2.1 mm × 100 mm, 1.7 µm) (Waters Corporation Milford, USA). A gradient mobile phase of (A) 2 mM ammonium acetate in 30: 70, methanol: MilliQ (B) 2 mM ammonium acetate in methanol at a flow rate of 0.30 mL min^{-1} was used.[1]

EOF was determined using combustion ion chromatography (CIC), with a combustion unit from Analytik Jena (Germany), a 920 Absorber Module, and 930 Compact IC Flex ion chromatograph from Metrohm (Switzerland). In brief, the extract (100 µL) was placed on a quartz boat and combusted in the furnace at 1000–1050 °C to convert organofluorine into HF, which was then adsorbed in MilliQ-water. The separation of anions was performed using an ion exchange column (Metrosep A Supp 5 – 150/4) with a carbonate buffer (64 mmol L^{-1} sodium carbonate and 20 mmol L^{-1} sodium bicarbonate) as eluent in isocratic elution. The concentration of F^{-} was determined by a conductivity detector.[1]

Suspect screening analysis was performed by using an Acquity UPLC system coupled with a quadrupole time-of-flight mass spectrometer (QTOF) (G2-XS, Waters Corporation, Milford, USA) in the electrospray negative and positive ionization modes. For negative mode, the separation of the compounds was performed by using an Acquity BEH C18 column (2.1 mm × 100 mm; 1.7 µm; Waters Corporation, Milford, USA) with the mobile phase (A) 2

mM ammonium acetate (in 30: 70, methanol: MilliQ) and (B) 2 mM ammonium acetate (in MeOH). The same LC conditions were applied for positve mode except that 2 mM ammonium acetate was replaced with 0.1% formic acid. A data-independent acquisition mode (MS^E) was used to obtain the precursor and fragment ions.[2, 3]

Conversion of PFAS concentration in F-equivalent concentration

To evaluate the extent of unidentified organofluorine, the measured PFAS concentrations of all target and suspect analytes (ng/mL PFAS) were converted to respective fluoride concentrations (ng/mL F) using the formula:

$$C_F = n_F \times \frac{MW_F}{MW_{PFAS_i}} \times C_{PFAS_i}$$

where C_F is the concentration of fluoride (ng/mL F) coming from the compound, n_F is the number of fluorine atoms in an analyte molecule, MW_F is the molecular weight of fluorine, MW_{PFASi} is the molecular weight of the analyte i, and C_{PFASi} is the concentration of analyte i (ng/mL $PFAS_i$). The amount of unidentified organofluorine was calculated as the difference between EOF and the target PFAS concentration after conversion into the fluoride equivalent.

Table S1. Mass spectrometric information for PFAS target analysis

Class	Abbreviation	Quantification ion (m/z)	Qualification ion (m/z)	Retention time (min)	Internal standard
PFCAs	PFBA	212.97/169	/	1.63	$^{13}C_4$-PFBA
	PFPeA	262.97/219	/	3.45	$^{13}C_3$-PFPeA
	PFHxA	312.97/269	312.97/118.9	5.31	$^{13}C_2$-PFHxA
	PFHpA	362.97/319	362.97/168.9	6.69	$^{13}C_4$-PFHpA
	PFOA	412.97/369	412.97/168.9	7.73	$^{13}C_4$-PFOA
	PFNA	462.99/419	462.99/219.0	8.12	$^{13}C_5$-PFNA
	PFDA	512.97/469	512.97/219.0	8.89	$^{13}C_2$-PFDA
	PFUnDA	562.97/519	562.97/268.9	9.55	$^{13}C_2$-PFUnDA
	PFDoDA	612.97/569	612.97/168.9	10.12	$^{13}C_2$-PFDoDA
	PFTrDA	662.9/619	662.9/168.9	10.61	$^{13}C_2$-PFDoDA
	PFTeDA	712.9/669	712.9/168.9	11.03	$^{13}C_2$-PFTeDA
	PFHxDA	812.9/769	812.9/168.9	11.53	$^{13}C_2$-PFHxDA
	PFOcDA	912.9/869	912.9/168.9	12.10	$^{13}C_2$-PFHxDA
PFSAs	PFBS	298.9/98.9	298.9/79.9	3.95	$^{13}C_3$-PFBS
	PFPeS	348.9/98.96	348.9/79.9	5.61	$^{18}O_2$-PFHxS
	PFHxS	398.9/98.9	398.9/79.9	6.84	$^{18}O_2$-PFHxS
	PFHpS	448.97/98.9	448.9/79.9	7.81	$^{13}C_4$-PFOS
	PFOS	498.97/98.9	498.9/79.9	8.61	$^{13}C_4$-PFOS
	PFNS	548.9/98.96	548.9/79.9	9.29	$^{13}C_4$-PFOS
	PFDS	598.97/98.9	598.97/79.9	9.88	$^{13}C_4$-PFOS
	PFDoDS	698.9/98.9	698.9/79.9	10.59	$^{13}C_4$-PFOS
PFAS precursors	4:2 FTSA	327/307	327.0/80.9	5.19	$^{13}C_2$-6:2 FTSA
	6:2 FTSA	427/407	427.0/80.9	7.68	$^{13}C_2$-6:2 FTSA
	8:2 FTSA	527/507	527.0/80.9	9.27	$^{13}C_2$-8:2 FTSA
	FHxSA	397.9/78	397.9/168.9	8.35	$^{13}C_8$-FOSA
	FOSA	497.9/78	497.9/168.9	9.88	$^{13}C_8$-FOSA

Table S2. Recoveries and MQL for PFAS target analysis

Compounds	MQL (ng/g dw)	Compounds	Average Recovery (%)	RSD (%)
PFBA	0.025	IS PFBA	66%	22%
PFPeA	0.025	IS PFPeA	72%	17%
PFHxA	0.025	IS PFHxA	85%	12%
PFHpA	0.025	IS PFHpA	88%	14%
PFOA	0.143	IS PFOA	85%	11%
PFNA	0.025	IS PFNA	82%	11%
PFDA	0.025	IS PFDA	98%	48%
PFUnDA	0.025	IS PFUnDA	75%	15%
PFDoDA	0.025	IS PFTeDA	62%	6%
PFTrDA	0.025	IS PFDoDA	53%	17%
PFTeDA	0.025	IS HFPO-DA	94%	9%
PFHxDA	0.025	IS PFBS	89%	9%
PFOcDA	0.025	IS PFHxS	78%	17%
PFBS	0.025	IS PFOS	65%	28%
PFPeS	0.025	IS FOSA	102%	7%
PFHxS	0.025	IS 6:2 FTSA	88%	46%
PFHpS	0.025	IS 8:2 FTSA	66%	22%
PFOS	0.117			
PFNS	0.025			
PFDS	0.025			
PFDoDS	0.025			
4:2 FTSA	0.025			
6:2 FTSA	0.150			
8:2 FTSA	0.025			
FHxSA	0.025			
FOSA	0.025			

Table S3. EOF concentrations in the alkaline and acid extractions

EOF (ng F/g dw)	Alkaline extraction	Acid extraction
A1	2030	0
A2	23990	166
A3	6440	45
A4	14260	326
A5	250	0
B1	2387	41
B2	73	131
B3	667	87
B4	447	258
C	245	0

Table S4. Target PFAS concentrations in the soil samples

Concentration (ng/g dw)	PFBA	PFPeA	PFHxA	PFHpA	PFOA	PFNA	PFDA	PFBS	PFHxS	PFOS	PFNS	PFDS	PFDoDS	FHxSA	FOSA	6:2 FTSA	8:2 FTSA
A1	4	14	5	2	9	2	3	<MQL	1	22	1	<MQL	2	3	7	12	11
A2	10	32	14	5	10	7	1	<MQL	1	53 (1)	<MQL	<MQL	3	6	8	134 (3)	83
A3	13	46	16	11	45 (1)	9	2	3	33	3244 (29)	19	7	11	35	41	35 (1)	39
A4	10	31	83	21	61	8	7	23	1674 (3)	20665 (11)	187	156	76	296	416	192 (3)	66
A5	3	7	1	1	2	1	1	<MQL	2	48	3	4	7	<MQL	1	1	<MQL
B1	0	1	1	<MQL	5	<MQL	<MQL	3	3	745 (25)	12	<MQL	3	33	37	11	2
B2	1	2	1	<MQL	2	<MQL	<MQL	<MQL	1	20	3	<MQL	<MQL	1	1	0	<MQL
B3	0	1	1	<MQL	1	<MQL	<MQL	<MQL	5	201 (4)	6	<MQL	1	29	3	1	<MQL
B4	0	0	0	<MQL	0	<MQL	<MQL	1	3	206 (3)	7	<MQL	<MQL	26	1	<MQL	<MQL
C	1	2	1	1	3	1	4	<MQL	5	115 (2)	6	108	<MQL	<MQL	<MQL	<MQL	<MQL

Note: PFUnDA, PFDoDA, PFTrDA, PFTeDA, PFHxDA, PFOcDA, PFPeS, PFHpS, and 4:2 FTSA were not detected.

The numbers in the brackets represent the concentrations of PFAS detected in the acidic extracts.

Table S5. Structure and the full name of PFAS screened using suspect screening in this work

Class	Full name	Abbreviation	Structure	Analogs detected
Class 1	Hydrogenated PFSA	H-PFSA	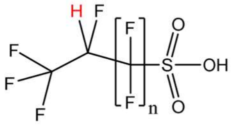	n = 6, 8
Class 2	n-PentaFluoro(5) Sulfide PFSA	F5S-PFSA	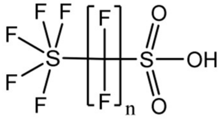	n = 6-10
Class 3	Ether-PFSA	E-PFSA	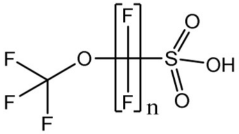	n = 8
Class 4	Ketone-PFSA	K-PFSA	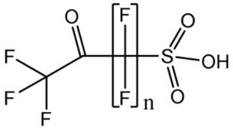	n = 6, 9, 10, 11
Class 5	Cl-PFSA	Cl-PFSA	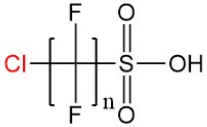	n = 8

Class 6	Unsaturated PFSA	UPFOS	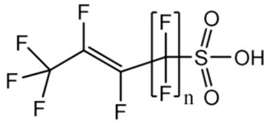	n = 5
Class 7	Perfluoroethylcyclohexane Sulfonate	PFECHS	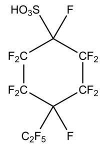	n.a.
Class 8	Perfluoroalkanesulfinate	PFASi	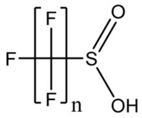	n = 6, 8
Class 9	n:2 fluorotelomer sulfonamide betaine	n:2 FTAB	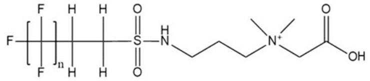	n = 4, 6, 8, 10, 12, 14
Class 10	N-Hydroxyethyl(HOE)dimethylAmmonio Propyl perFluoroAlkaneSulfonAmide	N-HOEAmP-FASA	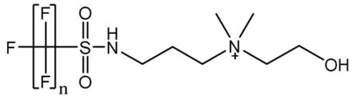	n = 6
Class 11	N-Hydroxyethyl(HOE)dimethylAmmonio Propyl perFluoroAlkane SulfonamidoEthanol	N-HOEAmP-FASE	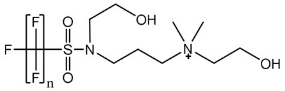	n = 6
Class 12	N-TrimethylAmmonioPropyl-perFluoroAlkaneSulfonAmide	N-TAmP-FASA	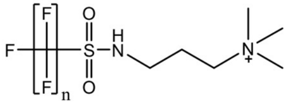	n = 6, 8
Class 13	n:2 fluorotelomer sulfoxide hydroxyammonium	n:2 FTSHA-sulfoxide	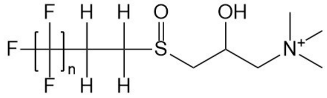	n = 6

Class 14	N-TrimethylAmmonioPropyl-Methyl perFluoroAlkaneSulfonAmide	N-TAmP-MeFASA	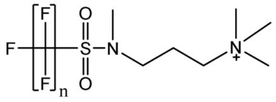	n = 6, 8
Class 15	PerFluoroAlkane Sulfonamido Amine	PFASaAm	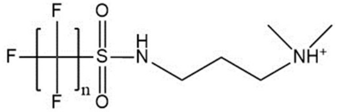	n = 6, 8
Class 16	N-SulfoPropyldimethylAmmonio Propyl perFluoroAlkaneSulfonAmide	N-SPAmP-FASA	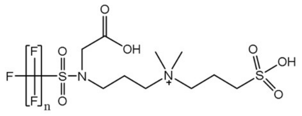	n = 6, 8
Class 17	N-CarboxyMethyldimethylAmmonioPropyl perFluoroAlkaneSulfonAmide	N-CMAmP-FASA	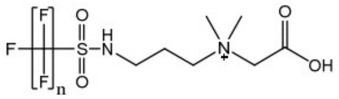	n = 6, 8

Table S6. Diagnostic fragments and reference standards of PFAS screened using suspect screening

Name	Formula	CL	Diagnostic fragment 1		Diagnostic fragment 2		Diagnostic fragment 3		Semi-quantification reference standards [a]
**ESI$^-$**	$[M–H]^-$								
H-PFOS	$C_8HF_{16}O_3S^-$	2b	460.9334	$C_8F_{15}SO_3^-$	79.9574	SO_3^-	98.9558	FSO_3^-	PFOS
H-PFDS	$C_{10}HF_{20}O_3S^-$	3b	560.9270	$C_{10}F_{19}SO_3^-$	79.9574	SO_3^-			PFDS
F5S-PFHxS	$C_6F_{17}O_3S_2^-$	2b	129.9542	$CF_2SO_3^-$	79.9582	SO_3^-	279.9446	$C_4F_8SO_3^-$	PFHxS
F5S-PFHpS	$C_7F_{19}O_3S_2^-$	3b	129.9542	$CF_2SO_3^-$	279.9446	$C_4F_8SO_3^-$			PFHpS
F5S-PFOS	$C_8F_{21}O_3S_2^-$	2b	379.9382	$C_6F_{12}SO_3^-$	279.9446	$C_4F_8SO_3^-$	479.9318	$C_8F_{16}SO_3^-$	PFOS
F5S-PFNS	$C_9F_{23}O_3S_2^-$	3b	279.9446	$C_4F_8SO_3^-$	479.9318	$C_8F_{16}SO_3^-$			PFNS
F5S-PFDS	$C_{10}F_{25}O_3S_2^-$	3b	479.9318	$C_8F_{16}SO_3^-$	579.9292	$C_{10}F_{20}SO_3^-$			PFDS
E-PFNS	$C_9F_{19}O_4S^-$	2b	79.9574	SO_3^-	98.9558	FSO_3^-	84.9906	CF_3O^-	PFNS
K-PFOS	$C_8F_{15}O_4S^-$	2b	79.9574	SO_3^-	98.9558	FSO_3^-	260.9461	$C_4F_7SO_3^-$	PFOS
K-PFUnDS	$C_{11}F_{21}O_4S^-$	3b	98.9558	FSO_3^-	360.9398	$C_6F_{11}SO_3^-$			PFDS
K-PFDoDS	$C_{12}F_{23}O_4S^-$	4	79.9574	SO_3^-	98.9558	FSO_3^-			PFDoDS
K-PFTrDS	$C_{14}F_{27}O_4S^-$	3b	79.9574	SO_3^-	98.9558	FSO_3^-			PFDoDS
Cl-PFOS	$C_8F_{16}O_3ClS^-$	3b	98.9558	FSO_3^-	79.9574	SO_3^-			PFOS
UPFOS	$C_8F_{15}O_3S^-$	3a	380.9766	$C_8F_{15}^-$	79.9574	SO_3^-	98.9558	FSO_3^-	PFOS
PFECHS	$C_8F_{15}O_3S^-$	3a	380.9766	$C_8F_{15}^-$	79.9574	SO_3^-	98.9558	FSO_3^-	PFOS
PFHxSi	$C_6F_{13}O_2S^-$	2b	318.9798	$C_6F_{13}^-$	168.9894	$C_3F_7^-$	82.9607	FSO_2^-	PFHxS
PFOSi	$C_8F_{17}O_2S^-$	2b	418.9734	$C_8F_{17}^-$	168.9894	$C_3F_7^-$	82.9607	FSO_2^-	PFOS
**ESI$^+$**	$[M+H]^+$								
4:2 FTAB	$C_{13}F_9H_{20}N_2SO_4^+$	2b	340.0049	$C_7H_7F_9NO_2S^+$	104.0708	$C_4H_{10}O_2N^+$	368.0362	$C_9F_9H_{11}SO_2N^+$	6:2 FTAB
6:2 FTAB	$C_{15}F_{13}H_{20}N_2SO_4^+$	2a	439.9985	$C_9H_7F_{13}NO_2S^+$	104.0708	$C_4H_{10}O_2N^+$	468.0298	$C_{11}F_{13}H_{11}SO_2N^+$	6:2 FTAB
8:2 FTAB	$C_{17}F_{17}H_{20}N_2SO_4^+$	2b	539.9921	$C_{11}H_7F_{17}NO_2S^+$	104.0708	$C_4H_{10}O_2N^+$	568.0234	$C_{13}F_{17}H_{11}SO_2N^+$	6:2 FTAB
10:2 FTAB	$C_{19}F_{21}H_{20}N_2SO_4^+$	2b	639.9857	$C_{13}H_7F_{21}NO_2S^+$	104.0708	$C_4H_{10}O_2N^+$	668.0170	$C_{15}F_{21}H_{11}SO_2N^+$	6:2 FTAB
12:2 FTAB	$C_{21}F_{25}H_{20}N_2SO_4^+$	2b	739.9793	$C_{15}H_7F_{25}NO_2S^+$	104.0708	$C_4H_{10}O_2N^+$	768.0107	$C_{17}F_{25}H_{11}SO_2N^+$	6:2 FTAB
14:2 FTAB	$C_{23}F_{29}H_{20}N_2SO_4^+$	3b	839.9729	$C_{17}H_7F_{29}NO_2S^+$	104.0708	$C_4H_{10}O_2N^+$			6:2 FTAB
N-HOEAmP-FHxSA	$C_{13}H_{18}F_{13}N_2O_3S^+$	2b	90.0913	$C_4H_{12}NO^+$	85.0886	$C_5H_{11}N^+$	116.1070	$C_6H_{14}NO^+$	6:2 FTAB

N-HOEAmP-FHxSE	$C_{15}H_{22}F_{13}N_2O_4S^+$	4	159.1492	$C_8H_{19}N_2O^+$	70.0655	$C_4H_8N^+$	455.9934	$C_9H_7F_{13}NO_3S^+$	6:2 FTAB
N-TAmP-FHxSA	$C_{13}H_{18}F_{13}N_2O_2S^+$	4	72.0808	$C_4H_{10}N^+$	130.1465	$C_7H_{18}N_2^+$	439.9985	$C_9H_7F_{13}NO_2S^+$	6:2 FTAB
N-TAmP-FOSA	$C_{15}H_{18}F_{17}N_2O_2S^+$	4	74.0964	$C_4H_{12}N^+$	130.1465	$C_7H_{18}N_2^+$	539.9920	$C_{11}H_7F_{17}NO_2S^+$	6:2 FTAB
6:2 FTSHA-sulfoxide	$C_{14}F_{13}H_{19}NO_2S^+$	2b	166.0896	$C_6H_{16}NO_2S^+$	116.1070	$C_6H_{14}NO^+$	453.0188	$C_{11}H_{10}O_2SF_{13}^+$	6:2 FTAB
N-TAmP-MeFHxSA	$C_{12}H_{16}F_{13}N_2O_2S^+$	2b	72.0808	$C_4H_{10}N^+$	130.1465	$C_7H_{18}N_2^+$	439.9985	$C_9H_7F_{13}NO_2S^+$	6:2 FTAB
N-TAmP-MeFOSA	$C_{14}H_{16}F_{17}N_2O_2S^+$	2b	74.0964	$C_4H_{12}N^+$	130.1465	$C_7H_{18}N_2^+$	539.9920	$C_{11}H_7F_{17}NO_2S^+$	6:2 FTAB
PFHxSaAm	$C_{11}F_{13}H_{14}N_2O_2S^+$	4	85.0886	$C_5H_{11}N^+$	70.0651	$C_4H_8N^+$	439.9985	$C_9H_7F_{13}NO_2S^+$	6:2 FTAB
PFOSaAm	$C_{13}F_{17}H_{14}N_2O_2S^+$	4	85.0886	$C_5H_{11}N^+$	70.0651	$C_4H_8N^+$	539.9920	$C_{11}H_7F_{17}NO_2S^+$	6:2 FTAB
N-SPAmP-FHxSA	$C_{14}H_{20}F_{13}N_2O_5S_2^+$	4	483.0406	$C_{11}H_{12}F_{13}N_2O_2S^+$	439.9985	$C_9H_7F_{13}NO_2S^+$	58.0651	$C_3H_8N^+$	6:2 FTAB
N-SPAmP-FOSA	$C_{16}H_{20}F_{17}N_2O_5S_2^+$	4	583.0342	$C_{13}H_{12}F_{17}N_2O_2S^+$	539.9920	$C_{11}H_7F_{17}NO_2S^+$	58.0651	$C_3H_8N^+$	6:2 FTAB
N-CMAmP-FHxSA	$C_{13}H_{16}F_{13}N_2O_4S^+$	4	173.1285	$C_8H_{17}N_2O_2^+$	104.0708	$C_4H_{10}O_2N^+$	411.9671	$C_7H_3F_{13}NO_2S^+$	6:2 FTAB
N-CMAmP-FOSA	$C_{15}H_{16}F_{17}N_2O_4S^+$	4	104.0708	$C_4H_{10}O_2N^+$	447.9988	$C_9H_3NF_{17}^+$	511.9607	$C_9H_3F_{17}NO_2S^+$	6:2 FTAB

[a] The selection of semi-quantification reference standards followed a previous study.[4] For ESI^-, legacy PFCAs were used as standards for PFASs with carboxylic acids functional group, PFSAs were used for PFASs with sulfonic acid functional group. For ESI^+, 6:2 FTAB was used as the reference standard.

Table S7. The proportion of PFAS left in the acid extraction

Compound	A1	A2	A3	A4	A5	B1	B2	B3	B4	C
6:2 FTAB	24%	9%	16%	13%	20%	10%	52%	31%	29%	0%
8:2 FTAB	23%	6%	14%	15%	24%	3%	43%	6%	18%	
10:2 FTAB	14%	4%	13%	12%		3%	40%	5%		
12:2 FTAB	11%	7%	14%	7%		9%				
N-HOEAmP-FHxSA	19%	18%	23%	44%	27%	10%	50%			
N-HOEAmP-FHxSE	16%	17%		45%			45%			
N-TAmP-FHxSA	16%	6%	5%	16%			34%		48%	
N-TAmP-FOSA									33%	
6:2 FTSHA-sulfoxide	6%	7%	12%	11%		16%				
N-TAmP-MeFHxSA	15%	11%	8%	25%		19%	42%	22%	21%	
N-TAmP-MeFOSA	10%	4%	11%	4%		15%			21%	
PFHxSaAm	28%	10%		41%	31%	24%				
N-SPAmP-FHxSA	30%	22%		23%	51%	30%	58%			
N-SPAmP-FOSA										
N-CMAmP-FHxSA		16%		15%						

Table S8. OF contribution from different proportion

Fluorine ng F/g dw	A1	A2	A3	A4	A5	B1	B2	B3	B4	C
Alkaline extraction EOF	2030	23990	6440	19250	250	2387	73	667	447	245
Acidic extraction EOF	0	166	45	326	0	41	131	87	258	0
Alkaline extraction target	63	227	2334	15458	53	553	21	160	158	163
Alkaline extraction SQ^+	693	7918	1533	2111	120	407	48	195	210	9
Alkaline extraction SQ^-	6	38	19	35	4	94	2	48	20	78
Acidic extraction Target	0	2	20	11	0	16	0	3	2	0
Acidic extraction SQ^+	168	310	77	284	27	77	37	49	78	3
Acidic extraction SQ^-	0	3	1	2	0	0	0	0	0	2
UOF	1101	15657	2501	1675	45	1281	95	300	238	-10
Proportion EOF explained (%)	46%	35%	61%	91%	82%	47%	53%	60%	66%	104%

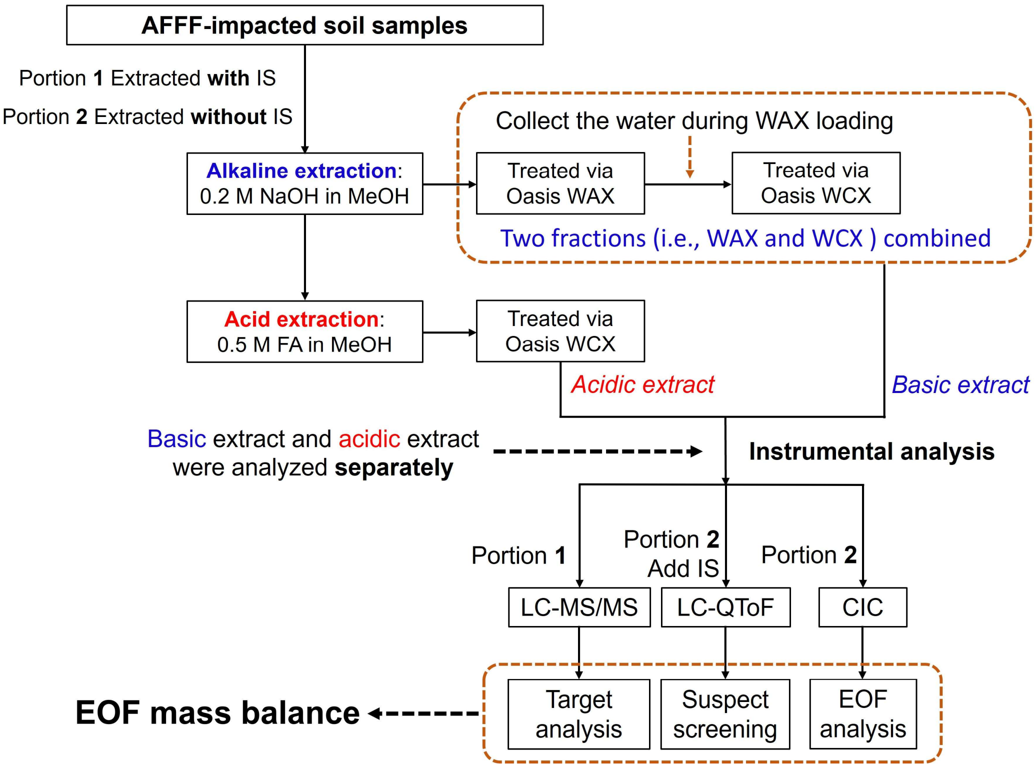

Figure S1. A workflow for sample extraction and analysis.

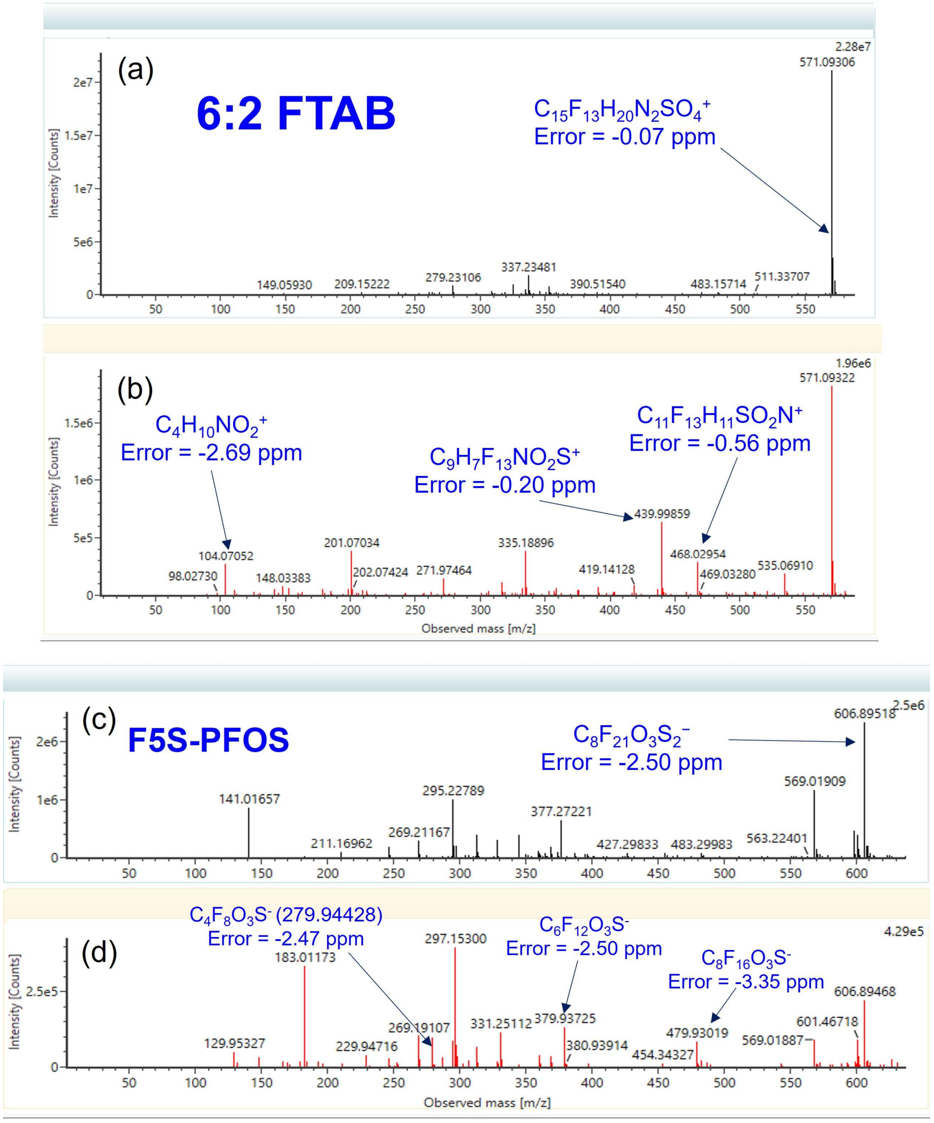

Figure S2. MS2 spectra for 6:2 FTAB in positive mode (a) low energy and (b) high energy and F5S-PFOS in negative mode (c) low energy and (d) high energy.

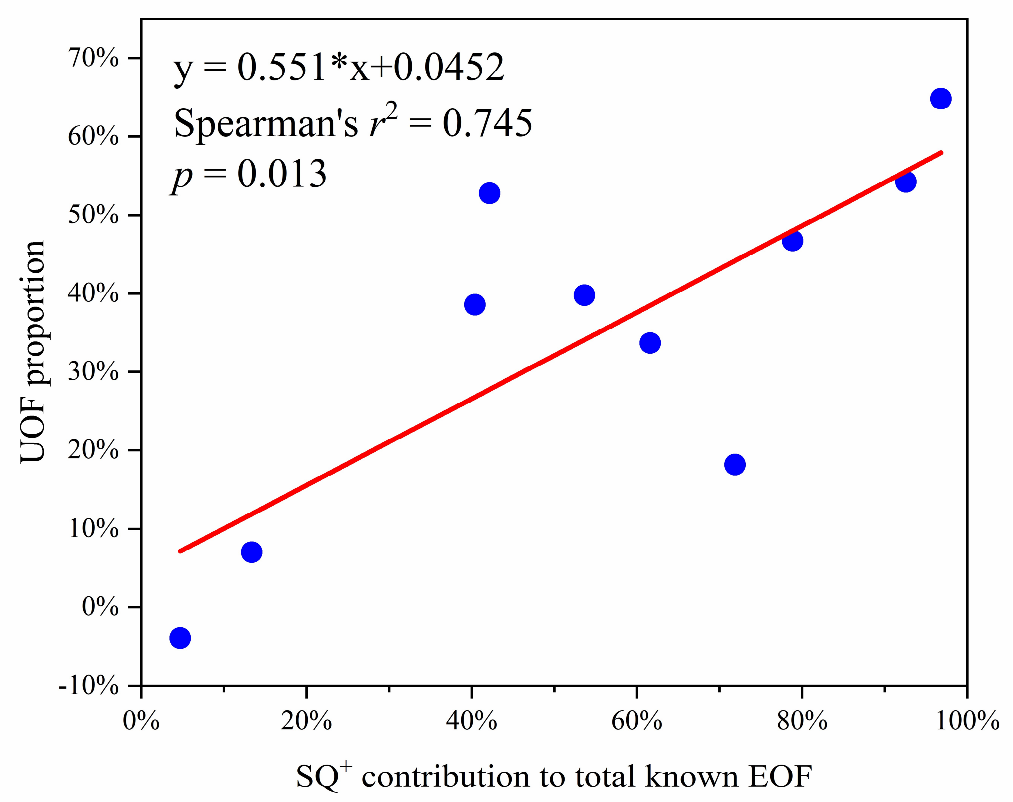

Figure S3. Relationship between SQ^+ to total known EOF and UOF proportion in soil samples.

References

1. Koch, A.; Karrman, A.; Yeung, L. W. Y.; Jonsson, M.; Ahrens, L.; Wang, T., Point source characterization of per- and polyfluoroalkyl substances (PFASs) and extractable organofluorine (EOF) in freshwater and aquatic invertebrates. *Environmental Science-Processes & Impacts* **2019,** *21* (11), 1887-1898.
2. Jiao, E.; Zhu, Z.; Yin, D.; Qiu, Y.; Kärrman, A.; Yeung, W. Y. L., A pilot study on extractable organofluorine and per-and polyfluoroalkyl substances (PFAS) in water from drinking water treatment plants around Taihu Lake, China: What is missed by target PFAS analysis? *Environmental Science: Processes & Impacts* **2022,** *24* (7), 1060-1070.
3. Koch, A.; Yukioka, S.; Tanaka, S.; Yeung, L. W. Y.; Karrman, A.; Wang, T., Characterization of an AFFF impacted freshwater environment using total fluorine, extractable organofluorine and suspect per- and polyfluoroalkyl substance screening analysis. *Chemosphere* **2021,** *276*, 130179.
4. Wang, X.; Yu, N.; Qian, Y.; Shi, W.; Zhang, X.; Geng, J.; Yu, H.; Wei, S., Non-target and suspect screening of per- and polyfluoroalkyl substances in Chinese municipal wastewater treatment plants. *Water Research* **2020,** *183*, 115989.
